# The reversibility and first-order nature of liquid–liquid transition in a molecular liquid

**DOI:** 10.1038/ncomms13438

**Published:** 2016-11-14

**Authors:** Mika Kobayashi, Hajime Tanaka

**Affiliations:** 1 Department of Fundamental Engineering, Institute of Industrial Science, University of Tokyo, 4-6-1 Komaba, Meguro-ku, Tokyo 153-8505, Japan

## Abstract

Liquid–liquid transition is an intriguing phenomenon in which a liquid transforms into another liquid via the first-order transition. For molecular liquids, however, it always takes place in a supercooled liquid state metastable against crystallization, which has led to a number of serious debates concerning its origin: liquid–liquid transition versus unusual nano-crystal formation. Thus, there have so far been no single example free from such debates, to the best of our knowledge. Here we show experimental evidence that the transition is truly liquid–liquid transition and not nano-crystallization for a molecular liquid, triphenyl phosphite. We kinetically isolate the reverse liquid-liquid transition from glass transition and crystallization with a high heating rate of flash differential scanning calorimetry, and prove the reversibility and first-order nature of liquid–liquid transition. Our finding not only deepens our physical understanding of liquid–liquid transition but may also initiate a phase of its research from both fundamental and applications viewpoints.

## Abstract (web summary)

The nature of the phenomenon of so-called ‘liquid-liquid transitions' in molecular liquids is a long-standing debate. Here, the authors demonstrate the reversibility and first-order nature of the liquid-liquid transition in triphenyl phosphite via flash differential scanning calorimetry.

Even for a single-component substance, there can be more than two liquid states^1^,^2^,^3^,^4^. The transition between these different liquid states is called ‘liquid–liquid transition (LLT)'. LLT is one of the most mysterious phenomena in liquid science and its presence and absence have often been debated for various systems. Since this problem is of fundamental importance in our understanding of the liquid state, LLT has kept attracting considerable attention.

The presence of LLT has been reported for both molecular systems (water^3^,^5^,^6^,^7^, triphenyl phosphite (TPP)^8^,^9^,^10^,^11^,^12^, n-butanol^13^ and possibly D-mannitol^14^) and atomic systems (sulfur^15^,^16^, phosphorus^17^, silicon^18^,^19^, germanium^20^ and Y_2_O_3_-Al_2_O_3_ (ref. 21)). Recently, LLT was also reported for metallic glass-formers^22^,^23^. However, none of these examples is free from controversy. The situation is more complicated for molecular liquids than for atomic liquids, since LLT always takes place in a supercooled state metastable against crystallization for molecular systems^4^. For atomic systems, on the other hand, the situation is better, since LLT often takes place in an equilibrium liquid state: For example, it was shown that liquid P shows a first-order like transition from P4 tetrahedra to polymereric P chain structure^17^ and liquid S transforms into different polymeric structures upon heating^15^,^16^.

One of the hottest and long-standing debates is on the nature of an unconventional amorphous state called ‘glacial phase' discovered by Kivelson and his coworkers^24^ for a molecular liquid, TPP. We note that TPP is one of the most well-studied molecular systems which are expected to have LLT. When TPP is kept at a low temperature near but still above the glass transition temperature *T*_g_ (∼204 K), a supercooled state of liquid 1 slowly transforms to an apparently amorphous state distinct from its ordinary glass state. Since this transformation occurs above *T*_g_ of liquid 1, 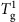, the final amorphous state must not be a glass state of liquid 1, glass 1. We showed that this phenomenon can naturally be explained by LLT from liquid 1 to a glass state of liquid 2, glass 2 (ref. 10). In this scenario, thus, the glacial phase is glass 2. In the following, for simplicity, we use the term ‘LLT' to express the transition between liquid 1 and liquid 2/glass 2 without distinguishing whether liquid 2 is in a liquid or glass state.

Besides the LLT scenario, many other explanations were also proposed for the nature of the glacial phase; for example, the glacial phase was interpreted as a mixture of glass 1 and nano-crystals^25^,^26^,^27^,^28^,^29^,^30^,^31^,^32^, a liquid crystal or plastic crystal^33^ and an unconventional crystal called defect-ordered crystal^34^. X-ray diffraction data of the glacial phase formed at a very low temperature show only broad amorphous peaks and no sharp Bragg peaks, indicating the absence of distinct translational order in the glacial phase^24^,^26^,^35^,^36^,^37^. This feature cannot be explained by the plastic-crystal scenario. The glacial phase prepared at a very low temperature does not exhibit distinct birefringence^10^,^38^,^39^, which cannot be explained by the liquid-crystal scenario. On the other hand, the glacial phase formed at a rather high temperature exhibits not only weak birefringence^24^,^38^,^39^ but also small Bragg peaks^30^. This can naturally be explained by the presence of nano-crystals in the glacial phase. Then the question is whether the glacial phase is primarily glass 2 or just a mixture of glass 1 and nano-crystals whose size decreases with a decrease in the annealing temperature, at which the glacial phase is formed. In the latter scenario, the absence of the Bragg peaks in the glacial phase formed at a very low temperature is ascribed to an extremely small size of nano-crystals.

Such debates may also originate from the counter-intuitive impression about LLT. According to classical liquid-state theory^40^, the liquid state can be described by a single order parameter, density *ρ*(**r**). Provided that a liquid is in a random disordered state, it is hard to accept the presence of two liquids with different densities intuitively. However, once we accept that we need an additional scalar order parameter besides density to describe the state of a liquid, LLT is no longer counter-intuitive and can be accepted naturally. On the basis of this idea and along the spirit of the pioneering works by Strässler and Kittel^41^ and Rapoport^42^, we proposed a two-order-parameter model of LLT^4^,^8^,^43^. In this picture, for example, LLT in atomic systems like sulfur and phosphorous^15^,^16^,^17^ can be explained by the distinct change in the locally favoured structures stabilized by chemical (or, covalent) bonding. Similarly, locally favoured structures can also be formed by directional hydrogen bonding for molecular liquids. According to our two-order-parameter model^4^,^8^,^9^, the order parameter governing LLT is the fraction of locally favoured structures, *S*, and then LLT is regarded as a gas–liquid-like transition of the order parameter *S*: Liquid 1 is a gas-like state with low *S* whereas liquid 2 is a liquid-like state with high *S*. Since locally favoured structures are created and annihilated independently, their number density is not conserved and thus *S* is a non-conserved scalar order parameter. Our recent X-ray scattering study^37^ revealed that upon LLT of TPP locally favoured structures whose size is a few nm are formed and its number density monotonically increases with time, and accordingly liquid 1 and liquid 2 can indeed be differentiated by the fraction of locally favoured structures *S*. Here we note that liquid 1 is a stable high-temperature liquid and liquid 2 is a low-temperature liquid, which usually exists in a glass state (glass 2) for TPP. This supports the two-order-parameter model of LLT^4^,^8^,^9^. However, the controversy has still remained due to the lack of direct experimental evidence for the presence of two liquid states and the reversibility of LLT. More importantly, no one has succeeded in avoiding nano-crystallization so far, which is due to an intrinsic difficulty associated with the fact that all the LLTs reported for molecular liquids such as TPP and water take place in a supercooled state below the melting point.

To firmly establish the LLT scenario, it is desirable to show the reversibility of LLT without suffering from any crystallization. For a heating rate <1 K s^−1^, however, the reverse LLT is hidden behind crystallization, even if it exists, since crystallization takes place immediately after the glass 2-to-liquid 2 transition during heating^10^. Terashima *et al.*^44^ observed an endothermic (heat absorbing) peak upon heating, which they attributed to the reverse LLT on the basis of the heating rate dependence of the onset temperature of the peak. However, if this is the reverse LLT, we should also observe a glass 2-to-liquid 2 transition during heating before glass 2 goes back to liquid 1, since the process of the reverse LLT should occur only in a liquid state and not in a glass state. But they reported only one endothermic peak. Furthermore, with a slow heating rate employed in the previous studies, it is impossible to access the entire reverse process from the glacial phase to liquid 1 due to the interference by crystallization. Thus, it is still unclear whether the peak is due to the melting of nano-crystals, the glass 2-to-liquid 2 transition and/or the reverse LLT process.

In this article, we aim at not only showing the reversibility of LLT but also confirming the coexistence of the two ‘liquid' states in the course of the transition in an unambiguous manner. These are crucial for proving that the transition takes place between two distinct liquid states. To this end, we apply ultra high-speed (flash) DSC (differential scanning calorimetry), which can provide a heating rate more than four orders of magnitude higher than that of conventional DSC (see Methods). This allows us to avoid crystallization upon heating and to directly access the reverse process of LLT without the interference by crystallization.

## Results

### Overall transition behaviours

**Figure 1:**
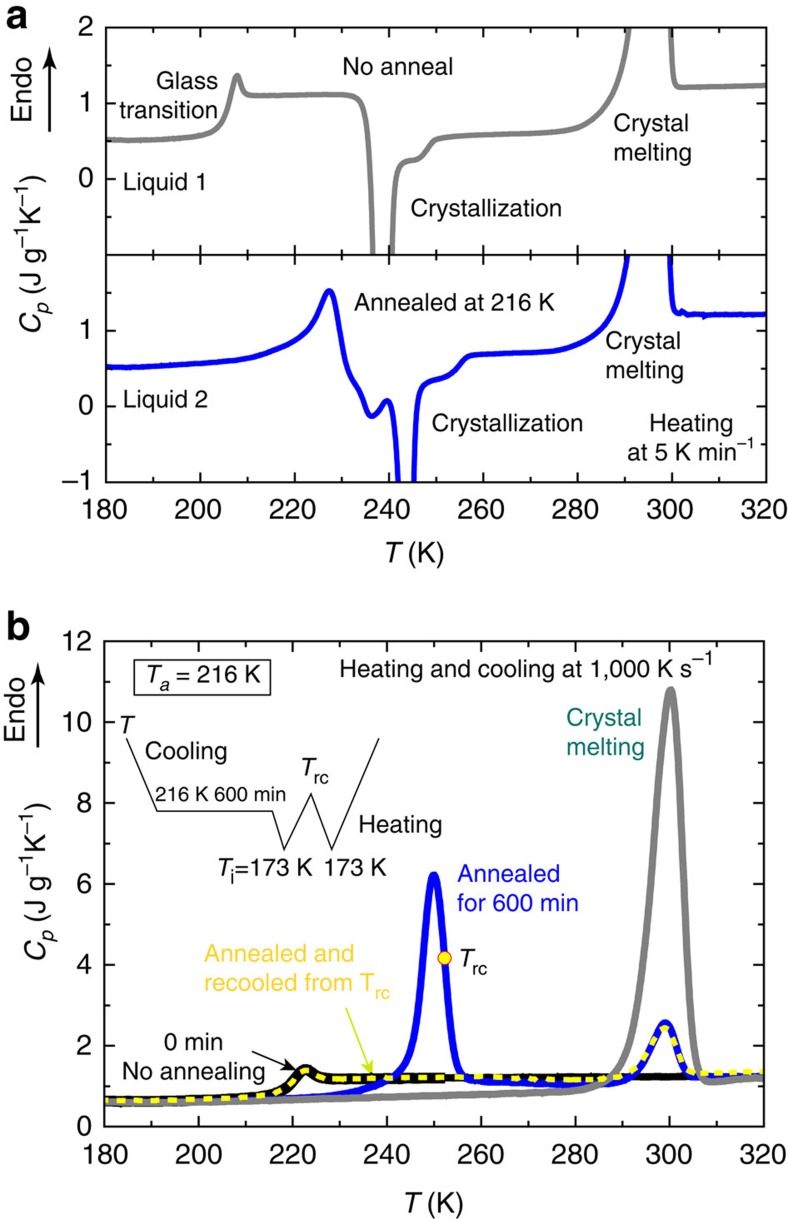
Comparison of DSC heat flow curves. (**a**) The results for the slow heating rate. The grey curve is a heating curve of liquid 1 without annealing and the blue curve is a heating curve of liquid 2 obtained after annealing for 600 min at 216 K. After the complete transformation from liquid 1 to the glacial phase (glass 2) by constant-temperature annealing, the glass 1-to-liquid 1 transition signal completely disappears and instead there appears an endothermic peak at higher temperature, which is then followed by the significant exothermic peak due to crystallization. This exothermic peak makes it difficult to clarify the origin of the endothermic process. (**b**) The results of flash DSC measurements. The black curve is obtained for a sample without annealing (liquid 1) and the blue curve is for a sample after annealing (the glacial phase, or glass 2). The yellow dashed curve is taken after re-cooled from a point *T*_rc_ in the endothermic peak (see the inset for the temperature protocol). The glass transition signal of liquid 1 is observed in the yellow dashed curve around 220 K, indicating that the glacial phase (glass 2) has already returned to liquid 1 during the endothermic process before reaching *T*_rc_. The grey curve is for a sample fully crystallized.

First we show calorimetric data obtained by the conventional DSC with a heating rate of 1/12 K s^−1^ in Fig. 1a (see Methods). The grey curve in the top panel is a DSC curve for liquid 1, which is obtained without annealing after liquid 1 is vitrified into its glassy state, glass 1. There we can see the glass 1-to-liquid 1 transition, whose onset is located around 204 K. The signal also has a large exothermic (heat releasing) peak due to crystallization around ∼240 K. On the other hand, the blue curve in the bottom panel shows a DSC heating curve for the glacial phase, which is prepared by annealing TPP for 600 min at 216 K until the transition is completed. We can see a change suggestive of the glass 2-to-liquid 2 transition, which starts around 210 K upon heating but is immediately followed by reverse LLT from liquid 2 to liquid 1 and crystallization (see below). The glass 2-to-liquid 2 transition is broader than the glass 1-to-liquid 1 transition, suggesting liquid 2 is less fragile than liquid 1 (ref. 10).

Next, we show DSC results obtained by the flash DSC with a heating rate of 10^3^ K s^−1^ in Fig. 1b (see Methods). The black curve is a DSC curve for liquid 1, which was obtained without annealing immediately after liquid 1 is vitrified into its glassy state, glass 1, as in the top panel of Fig. 1a. 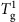 observed with a heating rate of 10^3^ K s^−1^ (∼217 K) is significantly higher than that observed with a slower heating rate of 1/12 K s^−1^ (∼204 K) by the conventional DSC (compare the black curve in Fig. 1b with the grey curve in the top panel of Fig. 1a). This is consistent with the general rule that the glass transition temperature increases with an increase in the heating rate. As shown in Fig. 1b (see the black curve), the DSC signal obtained with the ultra-high speed heating exhibits no exothermic heat due to crystallization during the heating process, unlike the case of the slow heating in Fig. 1a (see the grey curve). That is, there is no signature of crystal melting for a non-annealed sample (the black curve) in Fig. 1b, which is supposed to occur around 300 K (see the grey curve in Fig. 1a). This clearly shows that the ultra-high speed heating successfully avoids the occurrence of crystallization after the glass 1-to-liquid 1 transition. After TPP is annealed at 216 K for 600 min, on the other hand, the glass transition signal of liquid 1 completely disappears and instead a large endothermic peak appears around 250 K (see the blue curve in Fig. 1b) upon heating. This indicates that there is no liquid 1 (or glass 1) left in the glacial phase. This fact cannot be explained by the nano-crystal scenario, since it assumes that the glacial phase is a mixture of glass 1 and nano-crystals (see Supplementary Note 1 and Supplementary Figs 1–6 for further evidence against the nano-crystal scenario). Thus, we assign the glacial phase obtained by annealing to be glass 2.

**Figure 2:**
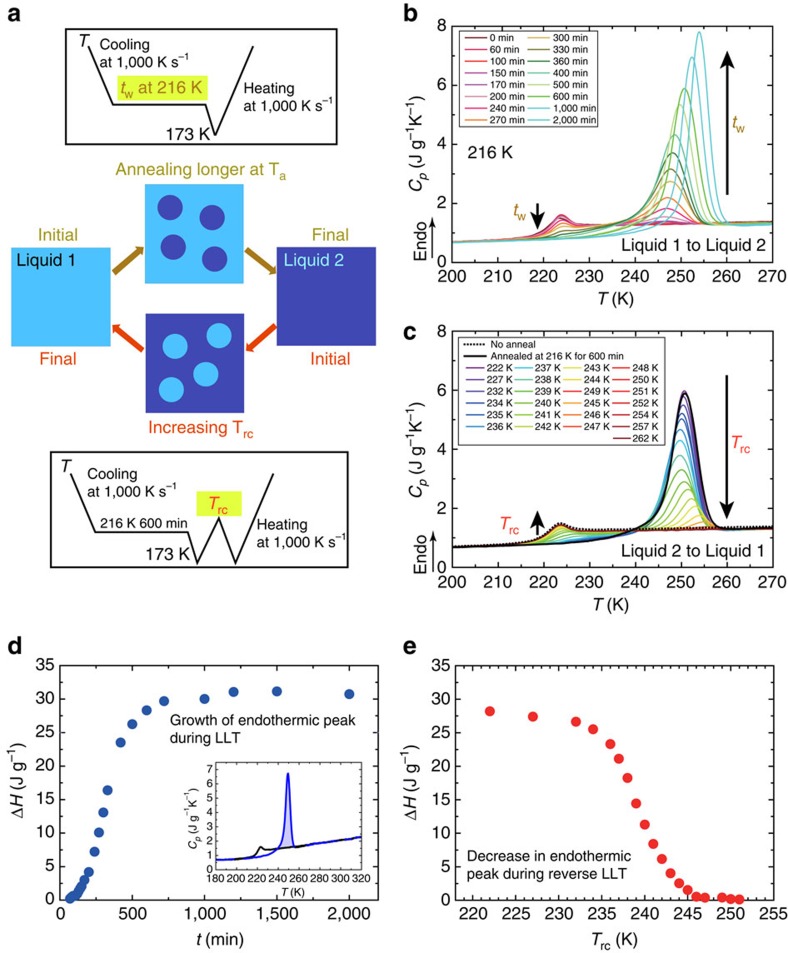
Forward and reverse LLT processes. (**a**) Schematic figures for forward LLT during annealing at *T*_a_ (top) and for reverse LLT as a function of the re-cooling temperature *T*_rc_ (bottom). The experimental protocols for them are also shown above and below the picture. (**b**) The forward LLT process from liquid 1 to liquid 2, probed by the reverse LLT process. The annealing time (*t*_w_-)dependence at *T*_a_=216 K. The numbers for lines with various colours denote *t*_w_. (**c**) The reverse LLT process from liquid 2 to liquid 1. The black dotted curve is for the sample without annealing and the black solid curve is for the sample annealed for 600 min at 216 K. The other curves are obtained for the second heating (see the protocol). The numbers for lines of various colours denote *T*_rc_. (**d**) The annealing time (*t*_w_-)dependence of the heat released by the reverse LLT upon heating for a sample annealed at 216 K. The inset illustrates how to estimate the transition heat by integration. (**e**) The re-cooling temperature (*T*_rc_-) dependence of the heat released upon heating for a sample annealed at 216 K for 600 min, in which LLT is completed. We can see that the onset of the reverse LLT, at which the endothermic heat starts to decrease, is located around 235 K. This temperature is located slightly above 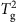.

In order to clarify what is happening at the endothermic peak, we employ the following special temperature protocol (see the inset in Fig. 1b and the bottom part of panel **a** of Fig. 2). First we anneal a sample at 216 K for 600 min, which completely transforms liquid 1 to the glacial phase, and then quench it to *T*_*i*_=173 K below *T*_g_. Next we heat the system until the temperature *T*_rc_ indicated by the yellow point on the blue curve, keep it at *T*_rc_ for a period of 0.1 s, and then cool it again from *T*_rc_ to *T*_*i*_=173 K below *T*_g_. Here we use the cooling and heating rate of 10^3^ K s^−1^. The second heating from *T*_*i*_ provides the yellow dashed DSC curve in Fig. 1b. We can clearly see the glass 1-to-liquid 1 transition signal in the yellow dashed curve. Furthermore, the perfect overlap of the glass transition signal between the black curve and the yellow dashed curve in Fig. 1b suggests that the glacial phase (or, glass 2) fully returns back to liquid 1 already much before crystal melting takes place in the heating process (more specifically, either before reaching *T*_rc_ in the first heating process or during 0.1 s kept at *T*_rc_). Thus, the endothermic peak around 250 K in the blue curve should not be associated with the crystals that should melt around 300 K.

We can see that the crystal melting behaviour around 300 K is almost perfectly the same between the blue curve and the yellow dashed curve (see Fig. 1b). This result clearly indicates that the crystals are formed exclusively during annealing and they are not affected by heating and cooling below *T*_rc_, suggesting that liquid 2 is prone to crystallization compared to liquid 1. We also note that the amount of the heat of fusion in the blue curve is much smaller than that in the grey curve, which is for a fully crystallized sample. This can be explained as follows: crystallization takes place preferentially in liquid 2/glass 2 domains, which are newly formed during annealing, but its glassy nature inhibits both nucleation and growth of crystals.

### The LLT scenario

We show experimental results on the forward and reverse LLT processes in much more detail (see the top part of Fig. 2a for the protocol and the resulting phase change process as a function of *t*_w_). The annealing time *t*_w_-dependence of the first heating curve is shown in Fig. 2b. The glass 1-to-liquid 1 glass transition signal becomes smaller with an increase in *t*_w_ and completely disappears for *t*_w_≥400 min, indicating that the liquid 1-to-glass 2 transition is completed around this annealing time. On the other hand, a new endothermic peak appears for *t*_w_≥200 min and continues to grow with an increase in *t*_w_. Another important fact is that after the endothermic peak the heat capacity of the liquid is the same as that of liquid 1, which can be seen from the fact that above 260 K all curves almost coincide with each other with the curve of *t*_w_=0 for liquid 1. This clearly indicates that the endothermic peak is associated with the transition from glass 2 to liquid 1. Furthermore, we can see in Fig. 2b that the endothermic peak position shifts to a higher temperature with an increase in *t*_w_. Figure 2d shows the *t*_w_-dependence of the total heat released by reverse LLT, which should be proportional to the amount of liquid 2 formed during *t*_w_.

We also show the *T*_rc_-dependence of the second heating curve in Fig. 2c (see the bottom part of Fig. 2a for the protocol and the schematic figure showing the resulting phase change process as a function of *T*_rc_). Here the sample is kept for 0.1 s at *T*_rc_ before re-cooling from *T*_rc_. With an increase in *T*_rc_, the endothermic peak becomes smaller and the glass 1-to-liquid 1 transition signal emerges and gradually becomes larger. Figure 2e shows the *T*_rc_-dependence of the heat released during the reverse LLT, which should be proportional to the amount of liquid 2 remaining after heated to *T*_rc_.

On the basis of these results, we discuss the origin of the new endothermic peak emerging after annealing at 216 K (see the blue curve in Fig. 1b). There are the following three possible origins for the endothermic peak appearing around 250 K: (i) the glass 1-to-liquid 1 transition, (ii) the glass 2-to-liquid 2 transition and (iii) the reverse LLT from liquid 2 to liquid 1. Whichever the transformed state contains liquid 1 or liquid 2, the system is initially in a glassy state and, thus, it should exhibit a glass transition signal upon heating before finally returning to liquid 1 (ref. 24). There is a difference in the heat flow level between before and after the endothermic peak, indicating the difference in the heat capacity *C*_*p*_ (see the blue curve in Fig. 1b). This is consistent with the occurrence of glass transition. However, we show below that the glass transition cannot be a primary origin of the endothermic peak.

First, we consider possibility (i) that the endothermic peak is due to the glass 1-to-liquid 1 transition. Although the position of the endothermic peak is significantly different from the glass transition peak of liquid 1 (*t*_w_=0), it alone does not immediately mean that the system is liquid 2 and not liquid 1. This is because ageing can generally shift the glass transition peak towards a higher temperature. Thus, even if the peak is due to the glass 1-to-liquid 1 transition, the peak position can depend on *t*_w_: the ageing of glass 1 should continuously shift the peak towards a higher temperature and increase the magnitude of the glass transition signal. Contrary to this expectation, however, Fig. 2b tells us that an increase in *t*_w_ reduces the signal of the glass-to-liquid transition and leads to the emergence of a new endothermic peak at a much higher temperature and the increase of its hight. This observation indicates that there are clearly two transitions with different origins. Thus, the presence of the two distinct transitions cannot be explained by scenario (i) based on the ageing of glass 1. This conclusion is also supported by the fact that after the transition (*t*_w_>400 min) the glass transition signal associated with liquid 1 component completely disappears.

Next, we consider possibility (ii) that the endothermic peak is mainly due to the glass 2-to-liquid 2 transition. The step-like change at a lower temperature is definitely associated with the glass 1-to-liquid 1 transition at least for a rather short annealing time *t*_w_. On the other hand, the endothermic peak appearing after annealing should be associated with liquid 2 formed during annealing. We note that the temperature shift of the peak towards a high temperature with an increase in *t*_w_ does not stop even after the transition is completed. However, the total heat released during the transition becomes constant after the completion of the transition (*t*_w_>1,000 min), as shown in Fig. 2d. The ageing of a glass should lead to the simultaneous increase in both the transition temperature and peak area. The lack of this feature indicates that the heat involved in the endothermic peak cannot be explained by the glass 2-to-liquid 2 transition alone, even taking the ageing effect into account. Furthermore, as shown in Fig. 1b, glass 2 has already returned to liquid 1 at *T*_rc_ upon heating, the endothermic peak should involve the reverse LLT (see also below for the further supporting evidence). Thus, we conclude that the endothermic peak is primarily not due to the glass 2-to-liquid 2 transition, although it should contribute partially.

Finally, we consider the remaining possibility (iii) that the endothermic peak should come mainly from the reverse LLT from liquid 2 to liquid 1. This scenario is strongly supported not only by the above-mentioned transformation of glass 2 to liquid 1 before reaching *T*_rc_ but also by the fact that the heat released by the reverse LLT (∼30 J g^−1^) (see Fig. 2d,e) is comparable to the heat absorbed by the forward LLT (∼25–27 J g^−1^). Here we note that the transition heat of the reverse LLT does not depend on the heating rate in the range of 500∼2,000 K s^−1^ and is almost constant within ±1%. This reflects that the transition is between the well-defined glass 2 state, which is almost uniquely determined by the annealing temperature and the annealing time, and the liquid 1 state. The difference in the transition heat between the reverse and forward LLT may come from the contribution of the glass 2-to-liquid 2 transition. The onset of the glass 2-to-liquid 2 transition marks the onset of the heat release (see below on the details of the glass transition behaviour). We note that the glass 2-to-liquid 2 transition provides the system with mobility, which is necessary for the reverse LLT to proceed.

In this reverse LLT scenario, we can explain the shift of the peak position towards a higher temperature with an increase in *t*_w_ as a consequence of the ageing of glass 2: Glass 2 becomes more stable and its glass transition temperature becomes higher with *t*_w_, leading to the shift of the onset of the reverse LLT towards a higher temperature. During annealing at *T*_a_, a system gradually transforms from liquid 1 to the glass state of liquid 2 (glass 2) with *t*_w_. Reflecting this, the total heat released during the reverse LLT should increase with an increase of *t*_w_. For *t*_w_>1,000 min, however, it becomes constant since LLT is completed, that is, the system almost perfectly becomes glass 2 (see Fig. 2b,d). This saturation indicates that the order parameter *S* in glass 2 becomes almost constant for *t*_w_>1,000 min. So we conclude that the transition behaviour shown in Fig. 2b consists of the step-like glass 2-to-liquid 2 transition and the endothermic peak due to the reverse LLT from liquid 2 to liquid 1 (see below on the separation of the two transitions).

Next, we consider the experimental results using the special temperature protocol (see the bottom part of Fig. 2a for the protocol and Fig. 2c for the results). In the above, we show that liquid 2 already fully returns to liquid 1 at the yellow point marked on the blue curve in Fig. 1b. Particularly, Fig. 2c shows that the glass transition signal associated with liquid 1 gradually recovers during the endothermic process with increasing *T*_rc_, clearly supporting the above-mentioned scenario that this endothermic peak is due to the reverse LLT process (liquid 2 → liquid 1). This is also consistent with the fact that the reverse LLT process from liquid 2 to liquid 1 should be an endothermic process since the forward LLT from liquid 1 to liquid 2 during isothermal annealing is an exothermic process^10^,^24^. We indeed confirm the amount of heat associated with the transition is about the same between the forward and reverse processes, as mentioned above. We can see in Fig. 2c that the peak position slightly shifts towards a higher temperature for higher *T*_rc_. This suggests that more stable parts of liquid 2 with higher *S* transform to liquid 1 at higher *T*_rc_.

### Glass transition behaviour and its link to the type of LLT

Now we focus on the glass transition behaviour taking place before the reverse LLT upon heating (see also Supplementary Note 2 and Supplementary Figs 7 and 8). The glass transition behaviours shown in Fig. 2b,c suggest that, in both processes of the forward and reverse LLT, the two disordered phases of liquid 1 and 2 coexist and transform reversibly with each other (see the schematic pictures in Fig. 2a). This is the first unambiguous evidence not only for the reversibility and the first-order nature of LLT in molecular liquids but also for the fact that the transition is between two distinct liquid states. The presence of the two glass transitions and the direct reversibility of the transition between the two liquid phases can be naturally explained by the LLT scenario, but not by the nano-crystal scenario.

**Figure 3:**
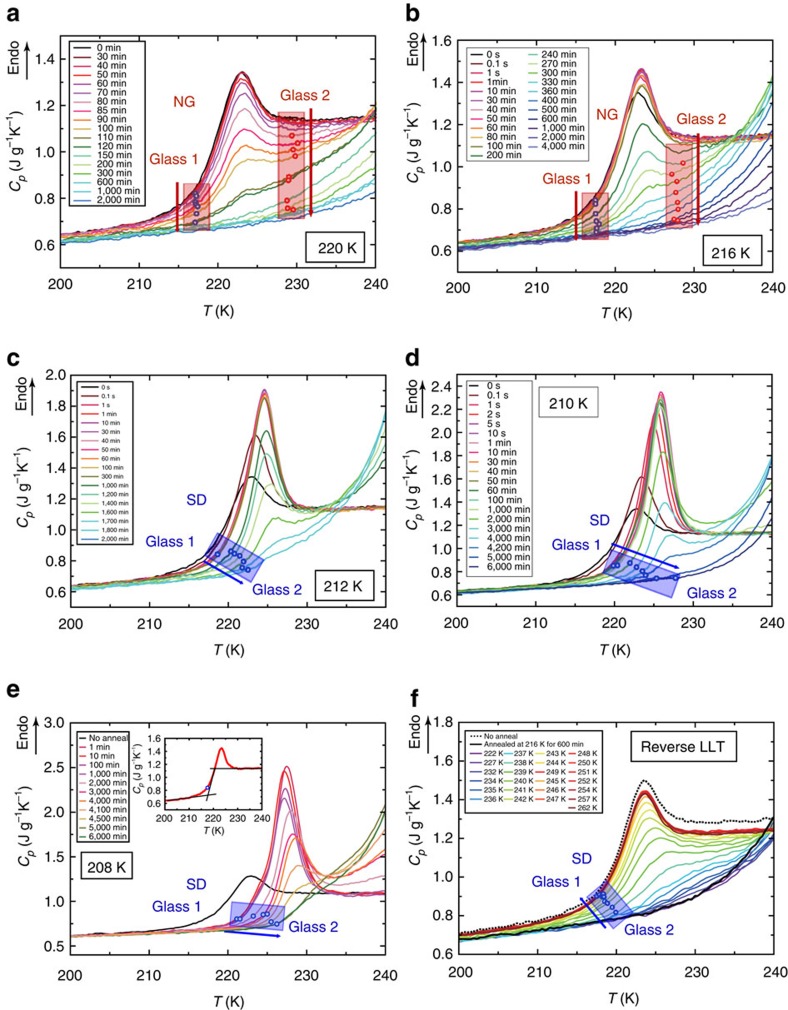
Glass transition behaviours during the process of forward and reverse LLT. (**a**,**b**) The temporal change in the glass transition behaviour during NG-type LLT observed at *T*_a_=220 K and 216 K, respectively. (**c**–**e**) The same observed during SD-type LLT observed at *T*_a_=212, 210, and 208 K, respectively. (**f**) The dependence of the glass transition behaviour as a function of *T*_rc_ in the reverse LLT process. The open circles show the onset temperatures of glass transition and the widths of the half transparent belts roughly represent possible errors in their determinations. An example of the estimation of the onset temperature of a glass transition is shown in the inset of **e**. The arrows indicate the directions of the increase in *t*_w_ for **a**–**e** and that in *T*_rc_ for **f**.

To be more quantitative, we identify the onset of the glass-to-liquid transition of the glass state obtained by various annealing time *t*_w_, as shown in Fig. 3 (see the inset of panel **e** on the determination of the onset of the glass transition). The glass transition behaviour provides crucial information on the type of LLT, that is, whether LLT is nucleation-growth (NG)-type or spinodal decomposition (SD)-type^4^. We note that NG-type LLT proceeds in a metastable state while accompanying nucleation of liquid 2 droplets in liquid 1, whereas SD-type LLT proceeds in an unstable state by a continuous transformation of liquid 1 to liquid 2.

When LLT proceeds above 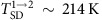, we have two sequential glass transitions upon heating. For *T*_a_=216 K, for example, we identify 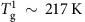 and 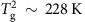 (see Fig. 3b). Interestingly, the positions of the onsets of the two glass transitions do not depend upon the annealing time within errors (±2 K). This is a characteristic feature of NG-type of LLT, reflecting that, for NG-type LLT, liquid 2 with the final order parameter value is nucleated in liquid 1 with the initial order parameter value and thus the order parameter changes discontinuously from that of liquid 1 to that of liquid 2. This coexistence of the two ‘liquid' phases during transformation can be regarded as a direct manifestation of LLT and its first-order nature^4^. Here it is worth mentioning that the glass 2-to-liquid 2 transition behaviour is not so clear compared with the glass 1-to-liquid 1 transition. The spinodal temperature, 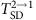, or the stability limit of liquid 2 against liquid 1 upon heating, is estimated to be around 235 K, which is located only slightly above the onset of the glass 2-to-liquid 2 transition. Thus, the glass transition does not complete at this temperature within a short time. This means that the plateau of *C*_*p*_ after the glass 2-to-liquid 2 transition never appears, making it difficult to observe a typical glass transition signal. For the reverse LLT to take place, the system needs to gain mobility. Once the system starts to gain mobility due to the glass 2-to-liquid 2 transition, the reverse LLT is immediately initiated. In other words, the glass transition and the reverse LLT almost simultaneously take place, making clear separation between the glass 2-to-liquid 2 transition and the reverse LLT intrinsically difficult.

When LLT proceeds below 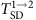 (∼214 K), on the other hand, we can see that there is only one glass transition, whose onset temperature continuously and gradually shifts from that of glass 1 to that of glass 2, as shown in Fig. 3c–e. We note that we analyse the data only after the ageing is completed (see Supplementary Fig. 9 and Supplementary Note 2). This glass transition behaviour is a characteristic feature of SD-type LLT, where the order parameter changes continuously with time^4^. Unlike the NG-type LLT, the reverse LLT starts immediately after the first glass transition step of the glass state, whose transition temperature is located between those of glass 1 and 2 in the process of LLT. Here it may be worth explaining why we may conclude that there is only one glass transition: Below 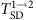 (∼214 K), the *C*_*p*_ exhibits a minimum following the glass-transition peak, but its value is larger than the heat capacity of liquid 1 (see the curve for *t*_w_=2,000 min at *T*_a_=210 K in Fig. 3d and the curves for *t*_w_≥2,000 min at 208 K in Fig. 3e). Considering that liquid 2 is in a more ordered state than liquid 1, the heat capacity of liquid 2 is expected to be smaller than that of liquid 1. Thus, the *C*_*p*_ minimum larger than that of liquid 1 should stem from an additional contribution to *C*_*p*_ from the reverse LLT. This means that the reverse LLT already starts before the completion of the glass 2-to-liquid 2 transition and there is no other glass transition. We note that such behaviour is never observed for the case of NG-type LLT (see Fig. 3a,b). This simultaneous occurrence of the glass transition and the reverse LLT is further supported by a clear two-step feature in the DSC curves for *t*_w_≥4,500 min at *T*_a_=208 K in Fig. 3e. The first step is the glass-to-liquid transition and the second one is the reverse LLT, unambiguously indicating that there are two distinct sequential transitions. Finally, we stress that these glass transition behaviours cannot be explained by the nano-crystal scenario, in which there should be only one glass transition from glass 1 to liquid 1 at 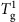.

**Figure 4:**
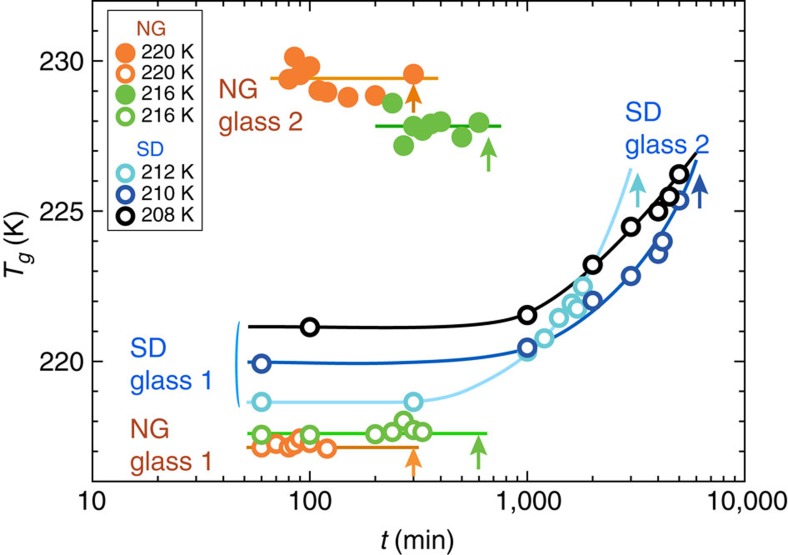
Behaviour of onset temperature of glass transition in the process of forward LLT. Each arrow indicates the time when the transition is completed for each annealing temperature. For NG-type LLT, the onset temperatures of the two glass transitions (glass 1 and 2) are both constant with *t*_w_, whereas for SD-type LLT there is only one glass transition and its onset temperature continuously increases with *t*_w_.

We compile all the data of the onset of the glass transition temperature in Fig. 4. There we can clearly see that for SD-type LLT below 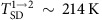 the onset temperature of the glass transition gradually and continuously shifts towards a high temperature with *t*_w,_ whereas for NG-type LLT above 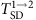 those of glass 1 and 2 stay almost constant as a function of *t*_w_ until the completion of the transformation, whose timing is indicated by the arrows in Fig. 4. This is fully consistent with the phenomenology of NG- and SD-type phase transformation^8^.

We stress that the above glass-transition behaviours are fully consistent with the characteristics of pattern evolution during LLT revealed by optical microscopy observation^10^. The microscopy observation suffered from a criticism stemming from a resolution problem: People may suspect that continuous evolution of smooth density fluctuations observed for SD-type LLT may be merely a consequence of that droplets are actually formed but too small to be optically resolved. Our DSC results clearly indicate that this is not the case and SD-type LLT of the continuous nature indeed takes place below 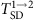. Thus, our finding strongly supports the physical picture of the two-order-parameter model of LLT^4^.

Finally, in the reverse LLT process, we can see that the onset temperature of the glass transition continuously shifts from that of liquid 2 to that of liquid 1, as shown in Fig. 3f. This indicates that the reverse LLT takes place via SD-type transformation. This is consistent with the fact that the process takes place almost immediately (<0.1 s) without an incubation time. This fast transformation process implies that the transformation takes place in a liquid state with fast dynamics, that is, far above the glass transition from glass 2 to liquid 2, 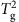. The onset temperature below which the transition heat starts to decrease is located around *T*_rc_∼235 K (see Fig. 2e). Thus, this *T*_rc_ marks the stability limit of liquid 2 against liquid 1 upon heating, that is, the temperature above which liquid 2 becomes unstable against liquid 1, that is, 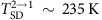. At ambient pressure, there is a rather large difference between 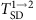 and 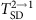, but this difference is expected to decrease with an increase in pressure and should disappear at the critical pressure *P*_c_. There the two spinodal temperatures 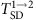 and 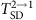 should merge to the critical temperature *T*_c_, which should be located above 235 K. From this, we can conclude that 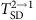 is located around 235 K for *T*_a_=216 K. This can also be confirmed in Fig. 3e (see the curve at *t*_w_=4,500 min), where we can see the glass transition and reverse LLT separately. We note that it is located slightly above 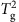 (∼228 K for *T*_a_=216 K at a heating rate of 10^3^ K s^−1^).

## Discussion

In summary, we reveal by ultra high-speed calorimetry that, upon heating, glass 2 that is formed by annealing liquid 1 at a low temperature for a long time, first transforms into liquid 2 via the glass transition and then almost simultaneously liquid 2 becomes liquid 1 via the reverse LLT. Our study not only shows the reversibility of LLT of TPP, but also its first-order nature from the coexistence of the two distinct liquid phases during LLT and the transformability between them. Furthermore, the glass transition behaviours of an intermediate state formed during LLT upon fast heating tell us that there are two types of LLT, NG-type and SD-type LLT, which are typical non-equilibrium dynamical processes of the first-order phase transition, respectively in its metastable and unstable state. We successfully reveal the discontinuous and continuous nature of the order parameter evolution for NG-type and SD-type LLT respectively from the glass transition behaviour of a transient state during LLT. This firm experimental confirmation of the first-order LLT transition in a single-component molecular liquid may initiate a new phase of theoretical and experimental research on the physical nature of this intriguing phase transition phenomenon and lead to a deeper understanding of the liquid state of matter. It may also contribute to the resolution of the controversy on LLTs of various systems such as water, which are also supposed to occur in a non-equilibrium metastable state^6^: our experimental method may be useful for isolating LLT from nano-crystallization for other systems.

## Methods

### Material

TPP (99.7% purity) was purchased from Across organics and used it without further purification. The melting point *T*_m_=297 K, whereas the glass transition temperature of liquid 1 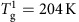 at a heating rate of 5 K min^−1^.

### Calorimetry measurements

We used an ultra high-speed DSC (Mettler-Toledo Flash DSC 1) and a conventional DSC (Mettler-Toledo DSC 1). In ultra high-speed DSC measurements, the sample mass was 20–50 ng, which was estimated for each sample by comparing the heat of fusion of a fully crystallized sample obtained by the flash DSC with that obtained by the conventional DSC. The fully crystallized sample was obtained by heating at 20 K min^−1^ from 173 K. In the forward and reverse LLT experiments, we used the protocol shown in Fig. 2a, where the cooling and heating rate were 1,000 K s^−1^, the lowest temperature was 173 K, a waiting time of 0.1 s was inserted between each scan for stabilization of the instrument. In conventional DSC measurements, the sample mass was 11.63 mg, the cooling and heating rates were 10 and 5 K min^−1^, respectively, and the lowest temperature was 173 K. All calorimetric measurements were performed under the N_2_ atmosphere.

### Data availability

The data that support the findings of this study are available from the authors upon request.

## Additional information

**How to cite this article:** Kobayashi, M. & Tanaka, H. The reversibility and first-order nature of liquid–liquid transition in a molecular liquid. *Nat. Commun.* **7**, 13438 doi: 10.1038/ncomms13438 (2016).

**Publisher's note:** Springer Nature remains neutral with regard to jurisdictional claims in published maps and institutional affiliations.

## Supplementary Material

Supplementary Information Supplementary Figures 1-9, Supplementary Notes 1-2 and Supplementary References
