# Supplementary material for: The reversibility and first-order nature of liquid–liquid transition in a molecular liquid
Source: Nat Commun. 2016 Nov 14;7:13438. doi: 10.1038/ncomms13438 (PMC5114579; doi:10.1038/ncomms13438)
Supplement: Supplementary Information — Supplementary Figures 1-9, Supplementary Notes 1-2 and Supplementary References [file ncomms13438-s1.pdf]

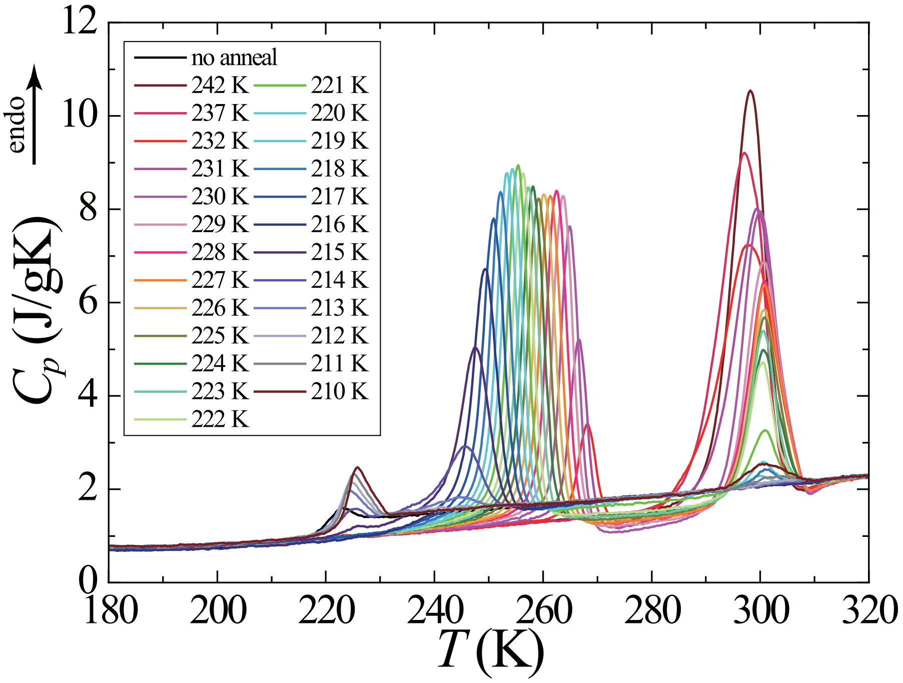

Supplementary Figure 1: The DSC curve of TPP annealed isothermally for 600 min at various T_a's upon heating at 1000 K/s. Endothermic peaks around 240-270 K and melting peaks of bulk crystals around 300 K are separated, suggesting that the origin of endothermic peak is not due to the melting of nano-crystals (see text).

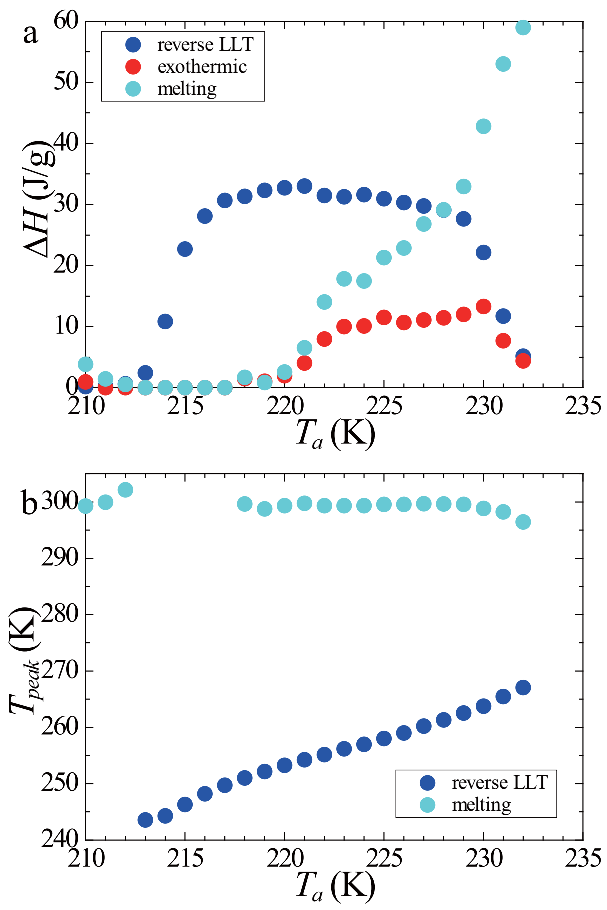

Supplementary Figure 2: **Characteristics of the transitions of TPP annealed isothermally for 600 min at various** T_{a}**'s, upon heating at 1000 K/s. a,** T_a-dependences of the transition heat of the reverse LLT, the exothermic signal appearing above $\sim$ 270 K due to crystallization, and the melting of crystal. **b,** T_a-dependences of the peak temperatures for the reverse LLT and melting.

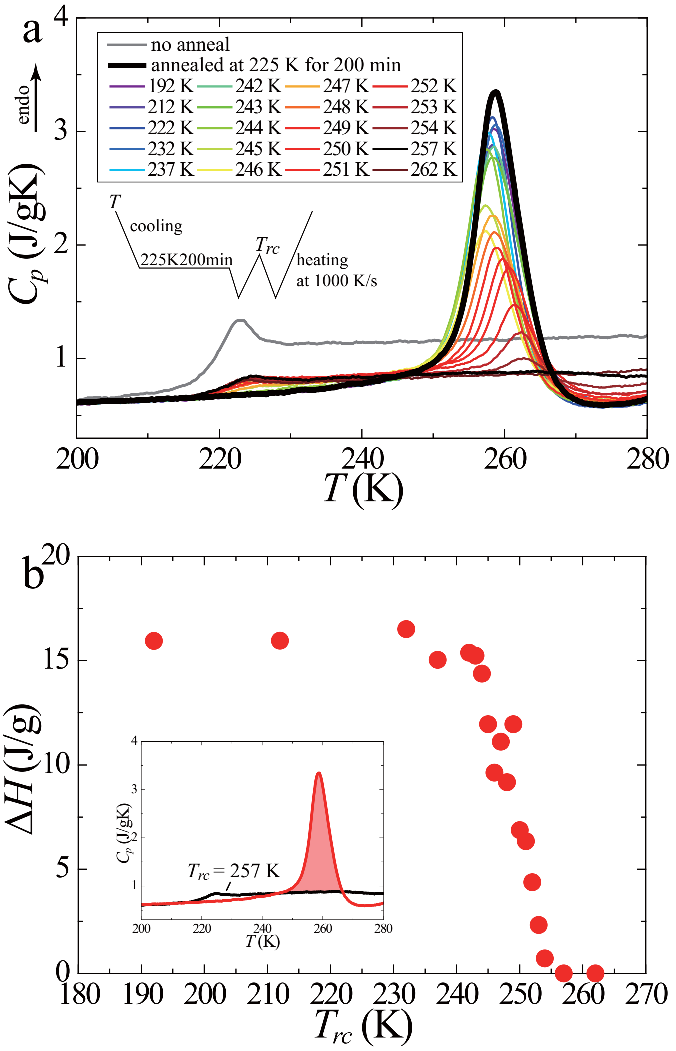

Supplementary Figure 3: The onset temperature of the reverse LLT for glass 2 formed at $T_{\mathrm{a}} = 225$ K. a, Reverse LLT processes upon second heating of samples, which are formed by annealing TPP at T_{a}=225 K for 200 min, then heated to T_{rc}, kept for 0.1 s there, and rapidly cooled to a low temperature. The temperature protocol and T_{rc} are shown in the inset. Note that only a part of the system returns to liquid 1 due to the crystallization. **b,** T_{rc}-dependence of the heat released upon heating estimated from the results in panel a. The inset explains how to estimate the total heat released. The onset of the reverse LLT is found to be located around 242 K.

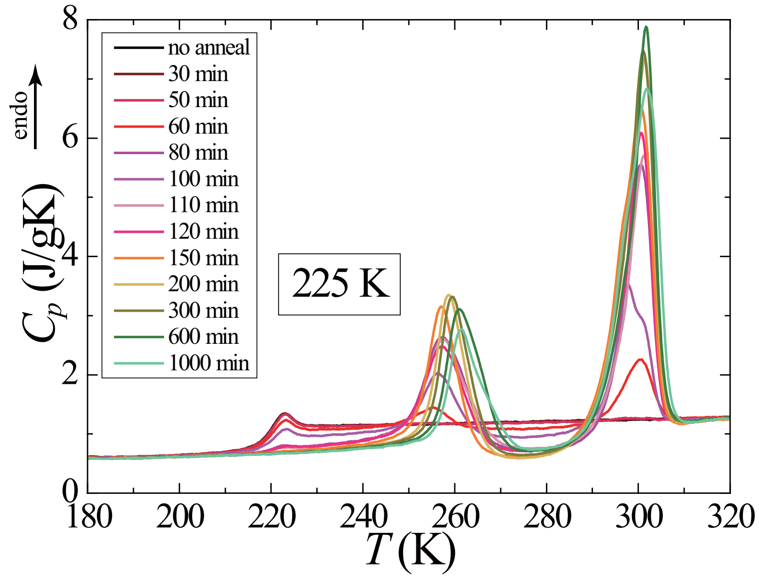

Supplementary Figure 4: **Annealing-time dependence of DSC heating curves of TPP annealed at 225 K.** The heating rate used in these experiments was 1000 K/s.

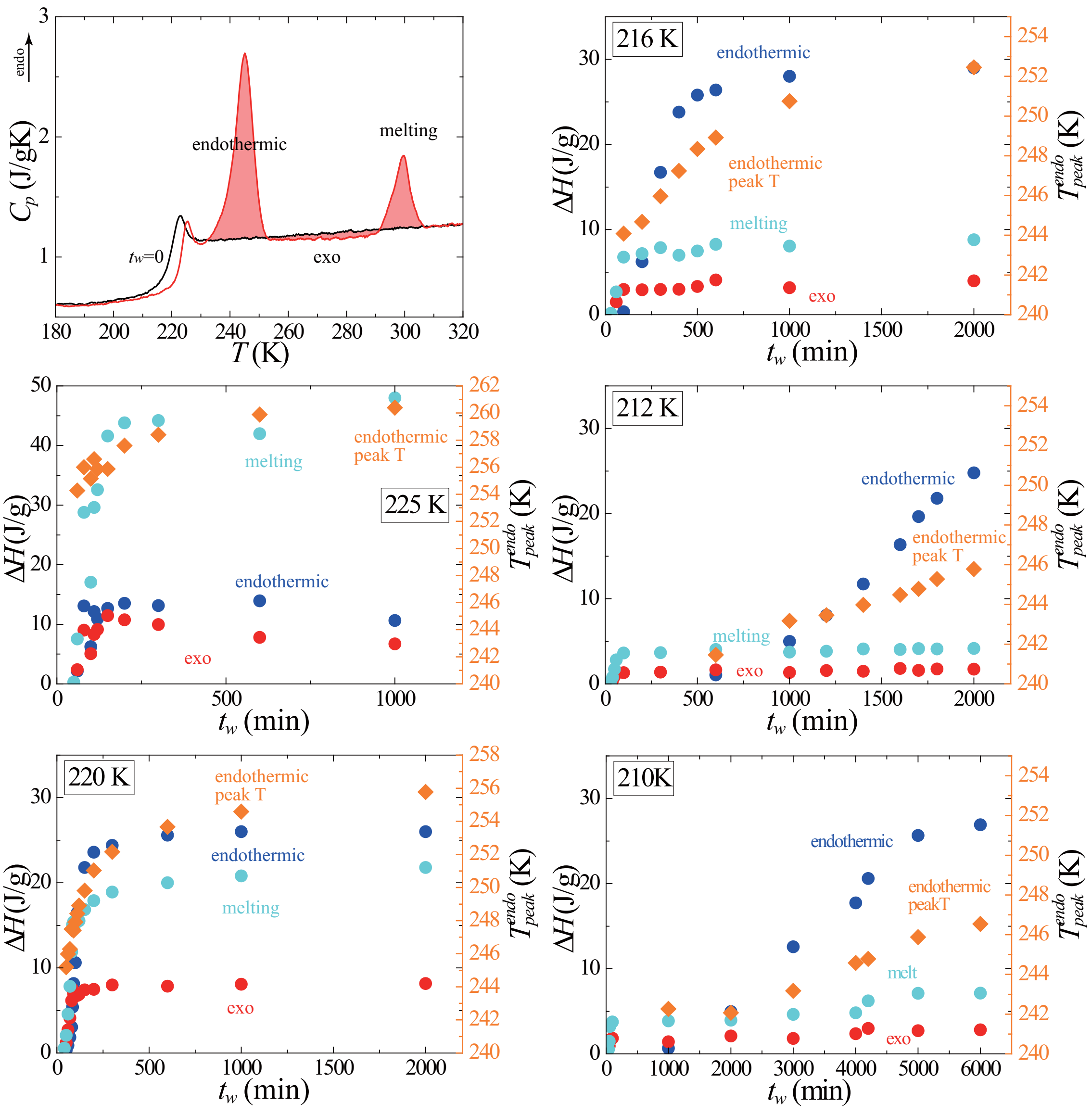

Supplementary Figure 5: The time evolution of the transition heat ΔH during LLT for various $T_{\rm a}$'s. We calculate ΔH by integrating the area of each heat process. We use a signal level at $t_w = 0$ as the base line of the integration and an example of our analysis is shown in the first top left panel.

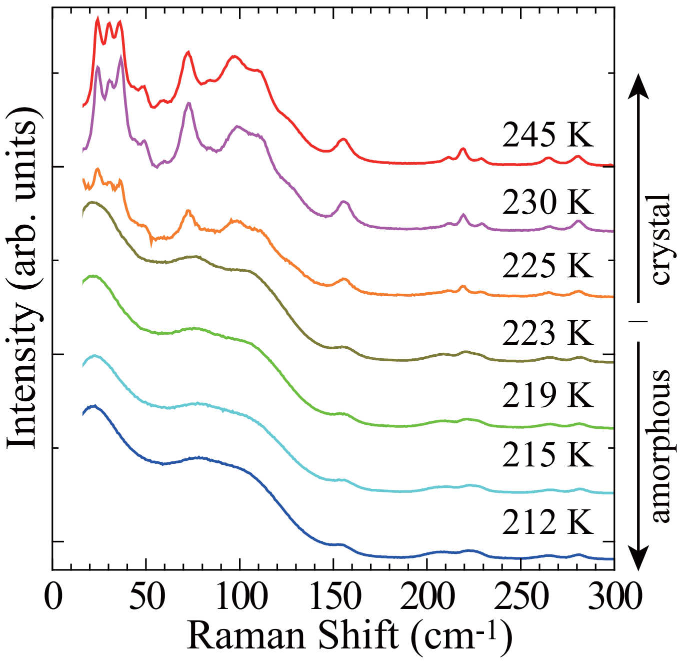

Supplementary Figure 6: **The annealing temperature T_a dependence of Raman spectra.** We use the polarized incident laser light (532 nm) for excitation and detect the scattered light of all polarizations. All measurements are made after LLT is completed. The signals above 225 K indicates the presence of crystals, whereas those below 223 K are typical amorphous Raman spectra and do not show any indication of the presence of crystals.

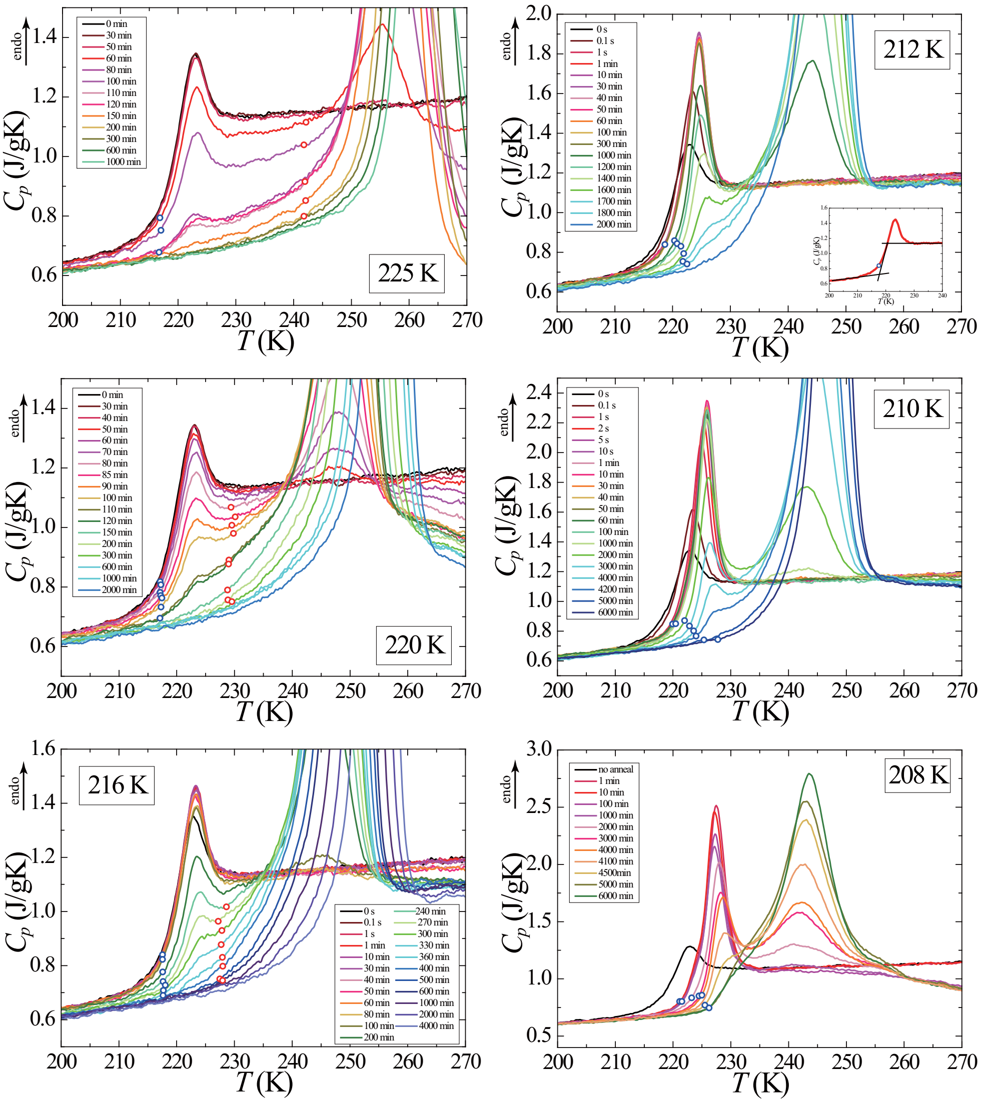

Supplementary Figure 7: **Annealing-time dependence of the glass transition and reverse LLT behaviours for six annealing temperatures.** Open circles denote the onset temperatures T_g. The $T_{\rm g}^1$ (blue circles) and $T_{\rm g}^2$ (red circles) do not change as a function of $t_{\rm w}$ above 214 K (NG-type), whereas $T_{\rm g}$ (blue circles) continuously changes below 214 K (SD-type). The inset in the panel of 212 K shows how to determine T_g.

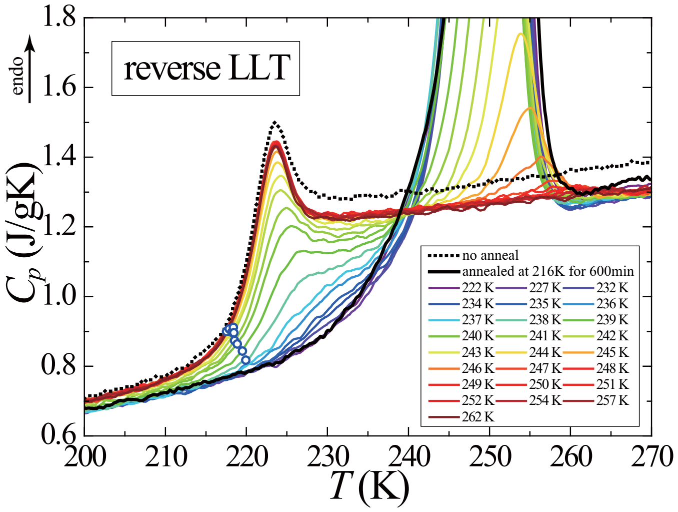

Supplementary Figure 8: $T_{\rm rc}$**-dependence of the glass transition behaviours during the reverse LLT.** In this case, we also see the gradual continuous change of $T_{\rm g}$ during the reverse LLT transition, which is suggestive of the SD-type nature of the transformation.

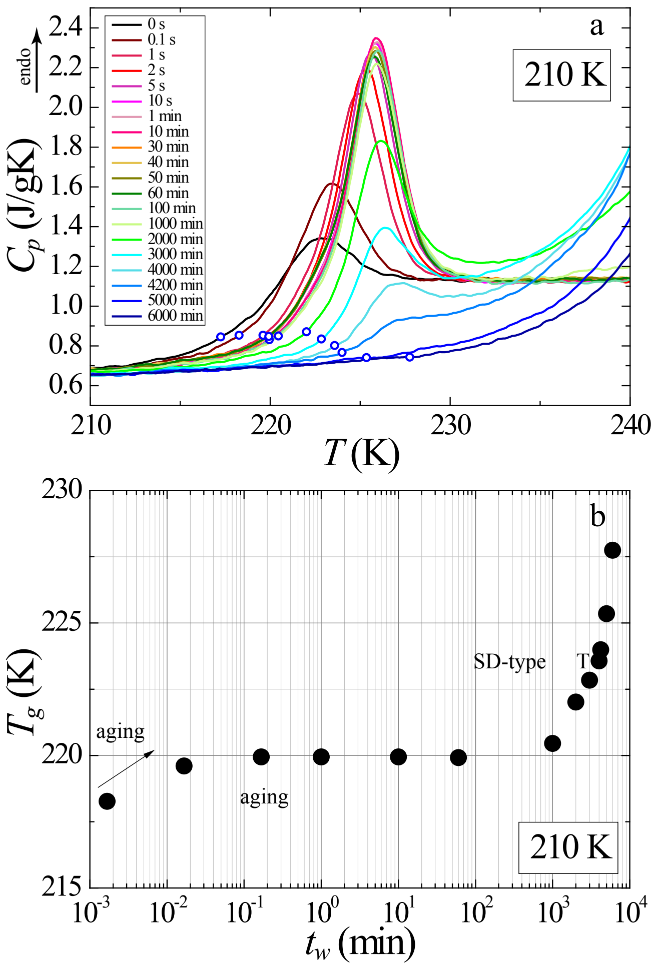

Supplementary Figure 9: **Temporal change in the glass transition behaviour during SD-type LLT at 210 K. a,** The temporal change in the glass transition behaviour observed at 210 K and the estimated onset temperatures of the glass transition (open circles). **b,** The time evolution of the onset temperature of the glass transition for a sample annealed at 210 K.

Supplementary Note 1: Difficulties of the nano-crystal scenario of the glacial phase

There have been long-standing arguments that the transition we focus here is nano-crystal formation [1–7]. In this scenario, the endothermic peak observed upon heating of the glacial phase should be due to the melting of nano-crystals. Although the endothermic peak is located at a temperature much lower than the melting point of bulk crystal, this might be explained by very small sizes of nano-crystals and their defective structures [4, 8]. If we assume this, the system can have two melting temperatures of crystals having very different sizes but with the same structure. This is a possible interpretation of the phenomenon. It is rather difficult to deny this scenario in a clear manner since nano-crystals are formed in a supercooed metastable liquid state anyway. This is the source of the long-lasting serious controversies on the nature of the glacial phase [9]. In the main text, we describe a few reasons why this scenario is difficult to explain our observation. Here we further discuss this possibility and show convincing experimental evidence that this transition cannot be explained by nano-crystal formation but should be LLT.

If we try to explain the endothermic peak by the nano-crystal scenario, we need to assign this peak as (i) the melting of very small crystals, (ii) the melting of a new type of crystal distinct from the known crystal, or (iii) the solid-state transition from an ordered to a disordered crystal. First of all, there is no sign of the glass transition associated with liquid 1 after the formation of the glacial phase (see Fig. 1b in the main text). In the nano-crystal scenario, the glacial phase is considered as a mixture of glass 1 and nano-crystals. Thus, the absence of the glass transition of liquid 1 means that the system is filled with nano-crystals without any amorphous parts. This is not consistent with a much smaller heat of fusion of the glacial phase than crystals (see Fig. 1b in the main text) and previous X-ray scattering experimental results [2, 10–12]. However, one may still argue that crystals are so disordered and thus the heat of fusion is much smaller than that of good crystals (see below). The melting of such small unstable nano-crystals might happen at a much lower temperature than T_m [4]. Then the endothermic peak might be the melting of such extremely small defective crystals. In this scenario, however, the gradual change of the glass transition temperature as a function of $T_{\rm rc}$ cannot be explained since there should be only liquid 1 that contributes to the glass transition. Furthermore, according to the X-ray scattering measurements, there is any indication of neither the formation of a new type of crystals, nor

the disappearance or position change of the Bragg peaks around 250 K. All these indicate that the above-mentioned scenarios based on nano-crystals cannot explain our DSC results.

Next we show the annealing temperature T_a-dependence of the heating curve of TPP samples after annealed for the same fixed duration of 600 min, in Supplementary Figure 1. Note that the transition is completed above $T_a = 216$ K before 600 min, whereas it is not completed below 216 K. Here we focus on the behaviours of the second transition appearing after the glass transition and the third transition around 300 K, which is the melting of crystals. We can see that the endothermic peak position of the second transition shifts towards a higher temperature with an increase in T_a. The peak eventually disappears for $T_{\rm a} > 232$ K and the highest observable peak temperature is located around 270 K. On the other hand, the melting of bulk crystals always takes place around 300 K irrespective of T_a.

We summarize the heat associated with phase transitions (the reverse LLT, crystallization during heating, and the crystal melting) in Supplementary Figure 2a and the peak temperatures of the reverse LLT and the crystal melting in Supplementary Figure 2b. The peak temperature of the endothermic peak of the reverse LLT monotonically increases with an increase in $T_{\rm a}$. Below $T_{\rm a} \sim 220$ K, the effect may be intrinsic, but above 220 K the peak temperature shift might be due to the presence of nano-crystals embedded in glass 2. The former is because there are few nano-crystals formed during annealing for $T_{\rm a} < 220$ K. On the other hand, the latter is because the amount of nano-crystals formed during the heating, which can be estimated by subtracting the heat released upon crystallization from the heat absorbed upon melting (see Supplementary Figure 2a), starts to increase above 220 K with an increase in $T_{\rm a}$ The increase in the onset temperature of the glass transition of liquid 2 and the reverse LLT for $T_{\rm a} < 220$ K may be due to the effects of ageing, since the ageing of glass 2 proceeds more quickly at a higher $T_{\rm a}$. In relation to this origin of the temperature shift, it should be noted that according to our previous X-ray scattering studies, the number density of locally favoured structures, or the order parameter S, does not depend on the annealing temperature $T_{\rm a}$. Since the $T_{\rm a}$-dependence of the endothermic position is not fully understood, however, we need further investigation to clarify its origin.

If we assume that the endothermic peak is due to the nano-crystal melting, the shift of the peak position towards a higher temperature can be explained by the larger size of crystals and/or their higher perfectness for higher T_a. This scenario suggests that the melting peak of nano-crystals should continuously shift towards that of bulk crystal. However, our

data shows that the endothermic peak cannot exist above 270 K, indicating its discontinuous jump to the melting peak of bulk crystal between $T_a = 232$ K and 237 K (see Supplementary Figure 1). This discontinuity between the endothermic peak and the melting peak of bulk crystals suggests that the endothermic peak is not due to nano-crystal melting.

We also note that the fact that the endothermic peak due to the reverse LLT disappears above 232 K indicates that only below this temperature LLT takes place. Thus, the binodal temperature of LLT is determined as $T_{\rm BN}$ ∼232 K. This value of $T_{\rm BN}$ is consistent with our previous estimation, $T_{\rm BN} \sim 230$ K [13, 14]. If we anneal a sample above this temperature, we have only crystallization phenomena and LLT cannot be induced.

The results shown in Supplementary Figure 1 also show clearly that the onset temperature of the reverse LLT increases with an increase in T_a, thus suggesting the increase of T_g^2 with T_a. We confirm that this is indeed the case. Supplementary Figure 3a shows the $T_{\rm rc}$-dependence of the endothermic signal coming from the reverse LLT for T_a=225 K. Supplementary Figure 3b plots the T_{rc}-dependence of the heat released upon heating estimated from the results in Supplementary Figure 3a. From this, we can estimate the onset of the reverse LLT to be located around 242 K, suggesting that $T_{\rm SD}^{2\to 1} \sim T_g^2 \sim 242$ K for glass 2 formed at T_a=225 K. In the main text we show that $T_{\rm SD}^{2\to 1} \sim 235$ K for $T_a \sim 216$ K. Thus, this result clearly indicates that T_g^2 and $T_{\rm SD}^{2\to 1}$ increase with an increase in T_a.

Next we show in Supplementary Figure 4 the time dependence of the DSC heating curve for TPP samples annealed at 225 K. At this temperature, the melting peak of bulk crystals around 300 K is very large compared to those for lower T_a and the amount of the crystals increases with an increase in the annealing time. On the other hand, the exothermic heat between the endothermic peak and the melting peak upon heating does not increase so much by increasing the annealing time, clearly indicating that the crystals which melt around 300 K is formed during LLT and not during heating. However, it is not reasonable to assume that nano-crystals and bulk crystals are formed simultaneously in the annealing process. Nano-crystals can be formed only under the situation that crystals cannot grow after nucleation. Extremely low mobility or internal frustration makes such a situation possible, but should make the formation of bulk crystals impossible. Thus, we conclude that it is difficult to have nano-crystals and bulk crystals at the same time in an ordinary situation.

Finally, we evaluate the transformation heat of each process: the endothermic peak due to the reverse LLT, the exothermic broad part due to crystallization, and the crystal melting

peak at several annealing temperatures (see Supplementary Figure 5). The time evolution of the endothermic heat reflects the process of the endothermic transition. It should be noted that the melting component grows before the endothermic peak grows. This trend is evident particularly at $T_{\rm a}$ below 216 K. For example, at $T_{\rm a} = 210$ K, the melting component appears around the annealing time of 30-60 min, whereas the endothermic peak appears around 1000 min. This fact strongly suggests that the endothermic transition and crystallization are the processes of essentially different nature. This also indicates the difficulty of the nano-crystal scenario and supports the LLT scenario.

In relation to the above, it should be noted that the crystal formation before the transition has been observed by neither light scattering nor X-ray scattering measurements. We show in Supplementary Figure 6 Raman scattering data at various T_a's, which are taken after LLT is completed. There are small peaks due to crystals in the data above $T_a = 225$ K, whereas such peaks are absent below 223 K and the spectra are typical amorphous signals, indicating the absence of crystals. These results indicate that crystals detected by our DSC in the process of the transformation during annealing should be unusually small, i.e., nano-crystals.

Supplemetary Note 2: Nucleation-growth and spinodal-decomposition-type LLT revealed by the glass-transition behaviours

According to our optical microscopy observation [13], there are two types of the dynamic processes in LLT of TPP: nucleation-growth (NG)-type and spinodal-decomposition (SD)-type LLT. This classification is based on the types of pattern evolution. We confirm the presence of these two types of LLT from the glass-transition behaviour of TPP during the LLT process, as discussed in the main text. We stress that the glass transition behaviour is specific to a liquid state. The existence of the two glass transition temperatures for a single-component liquid and the presence of two types (NG-type and SD-type) temporal changes of the glass transition temperatures strongly support that the transition is indeed LLT and the endothermic peak observed on heating is the reverse LLT.

Each feature of NG-type and SD-type LLT appears in the behaviour of the glass transition temperature, $T_{\rm g}$. Supplementary Figure 7 is the annealing time dependence of the glass

transition temperature for several annealing temperatures. We estimate $T_{\rm g}$ as the onset temperature of the glass transition upon heating. The method to estimate T_g is shown in the inset of the panel of $T_{\rm a} = 212$ K. The $T_{\rm g}$ determined in this way is shown by an open circle on each curve. We analyse only the data after ageing of liquid 1 to see the change of the glass transition temperature due to LLT alone. Above 216 K, T_g shows almost no change with the progress of LLT, indicating that this glass transition is always that of liquid 1. The magnitude of the glass transition step, or the amount of liquid 1, monotonically decreases with the annealing time, reflecting the nucleation and growth of liquid 2 domains in the sample, which was observed with optical microscopy [13]. This is the typical behaviour expected for the NG-type LLT. On the other hand, T_g continuously shifts towards a higher temperature with the waiting time for $T_{\rm a}$ below 212 K, suggesting that the transition from liquid 1 to liquid 2 is continuous. This behaviour is consistent with SD-type LLT. These results clearly indicate that the dynamical process of LLT can be classified into NG-type and SD-type (see also Fig. 4 in the main text). This is fully consistent with our microscopic observation of pattern evolution [13], strongly supporting the LLT scenario [15].

Supplementary Figure 8 shows the $T_{\rm rc}$-dependence of the glass transition behaviours during the reverse LLT. The gradual continuous change of $T_{\rm g}$ during the reverse LLT transition suggests that this process may be SD-type transformation, which is consistent with its rather rapid transformation.

Finally we show the effects of ageing on the onset temperature of the glass transition by taking the data of $T_{\rm a} = 210$ K as an example. Supplementary Figure 9a shows the DSC curves in the glass transition region for $T_{\rm a} = 210$ K, whereas Supplementary Figure 9b plots the estimated onset temperature against the annealing time $t_{\rm w}$. We can see that the initial increase of $T_{\rm g}$ is due to the ageing of glass 1. The time region of the constant $T_{\rm g}$ of glass 1 indicates that the ageing of glass 1 is completed after 10^{-1} min. The final continuous increase after 10^3 min is due to SD-type LLT, which can be seen in Supplementary Figure 9b.

Supplementary References

[1] Hédoux, A., Guinet, Y. & Descamps, M. Raman signature of polyamorphism in triphenyl phosphite. *Phys. Rev. B* **58**, 31 (1998).

[2] Hédoux, A., Hernandez, O., Lefebvre, J., Guinet, Y. & Descamps, M. Mesoscopic description of the glacial state in triphenyl phosphite from an x-ray diffraction experiment. *Phys. Rev. B* **60**, 9390 (1999).

[3] Hédoux, A., Derollez, P., Guinet, Y., Dianoux, A. J. & Descamps, M. Low-frequency vibrational excitations in the amorphous and crystalline states of triphenyl phosphite: A neutron and Raman scattering investigation. *Phys. Rev. B* **63**, 144202 (2001).

[4] Hédoux, A., Denicourt, T., Guinet, Y., Carpentier, L. & Descamps, M. Conversion of the glacial state into the crystal in triphenyl phosphite. *Solid State Commun.* **122**, 373–378 (2002).

[5] Hédoux, A. *et al.* A description of the frustration responsible for a polyamorphism situation in triphenyl phosphite. *J. Non-Cryst. Solids* **307**, 637–643 (2002).

[6] Hédoux, A. *et al.* Micro-structural investigations in the glacial state of triphenyl phosphite. *J. Non-Cryst. Solids* **352**, 4994–5000 (2006).

[7] Baran, J., Davydova, N. A. & Drozd, M. Polymorphism of triphenyl phosphite. *J. Chem. Phys.* **140**, 104512 (2014).

[8] Alba-Simionesco, C. & Tarjus, G. Experimental evidence of mesoscopic order in the apparently amorphous glacial phase of the fragile glass former triphenylphosphite. *Europhys. Lett.* **52**, 297–303 (2000).

[9] Tanaka, H. Importance of many-body orientational correlations in the physical description of liquids. *Faraday Discuss.* **167**, 9–76 (2013).

[10] Derollez, P. *et al.* Structural and microstructural description of the glacial state in triphenyl phosphite from powder synchrotron X-ray diffraction data and Raman scattering investigations. *J. Mol. Struct.* **694**, 131–138 (2004).

[11] Mei, Q., Ghalsasi, P., Benmore, C. J. & Yarger, J. L. The local structure of triphenyl phosphite studied using spallation neutron and high-energy X-ray diffraction. *J. Phys. Chem. B* **108**, 20076–20082 (2004).

[12] Murata, K. & Tanaka, H. Microscopic identification of the order parameter governing liquid-

liquid transition in a molecular liquid. *Proc. Nat. Acad. Sci. USA* **112**, 5956–5961 (2015).

[13] Tanaka, H., Kurita, R. & Mataki, H. Liquid-liquid transition in the molecular liquid triphenyl phosphite. *Phys. Rev. Lett.* **92**, 025701 (2004).

[14] Kurita, R. & Tanaka, H. Critical-like phenomena associated with liquid-liquid transition in a molecular liquid. *Science* **306**, 845–848 (2004).

[15] Tanaka, H. General view of a liquid-liquid phase transition. *Phys. Rev. E* **62**, 6968 (2000).
